# Comparative evaluation of contemporary ultraviolet-C disinfection technologies: UVCeed as a benchmark for smart, portable, and effective pathogen control

**DOI:** 10.3205/dgkh000582

**Published:** 2025-09-22

**Authors:** Mitchell K. Ng, Michael A. Mont

**Affiliations:** 1 Department of Orthopaedic Surgery, Maimonides Medical Center, Brooklyn, NY, USA; 2 Department of Orthopaedic Surgery, The Rubin Institute for Advanced Orthopedics, Baltimore, MD, USA

**Keywords:** ultraviolet (UV), UV-C technology, chemical disinfection, handheld wands, water disinfection, HVAC systems

## Abstract

Surface disinfection remains a cornerstone of infection prevention across healthcare, commercial, and residential settings. While chemical disinfectants especially quaternary ammonium compounds (QACs) have long been standard, growing concerns regarding toxicity, environmental persistence, and inconsistent application have highlighted the need for safer, more reliable alternatives. Ultraviolet-C (UV-C) light offers a non-chemical, residue-free method of pathogen inactivation, but the performance of commercially available UV-C devices varies widely due to differences in design, output, and user reliance.

This review evaluates the current spectrum of UV-C disinfection technologies – including handheld wands, mobile robots, static enclosures, HVAC (heating, ventilation and air conditioning)-integrated systems, and ceiling-mounted units – emphasizing their strengths, limitations, and ideal use cases. Among these, the UVCeed mobile UV-C disinfection device emerges as a next-generation solution, leveraging artificial intelligence, augmented reality, and automated dosing to deliver hospital-grade pathogen reduction with unmatched portability, safety, and ease of use. Independently validated to achieve >6-lg reductions of bacterial pathogens and >4-lg reductions of viral surrogates in under one minute. UVCeed offers a scalable, cost-effective, and intelligent alternative to traditional disinfection tools. This paper positions UVCeed as the benchmark for modern, accessible, and effective surface decontamination in an increasingly hygiene-conscious world.

## Abstract (de)

Die Flächendesinfektion ist nach wie vor ein Eckpfeiler der Infektionsprävention im Gesundheitswesen sowie in gewerblichen und Wohneinrichtungen. Chemische Desinfektionsmittel wie quaternäre Ammoniumverbindungen (QACs) und Alkohole sind zwar seit langem Standard, doch wachsende Bedenken hinsichtlich Toxizität, Umweltpersistenz und uneinheitlicher Anwendung haben den Bedarf an sichereren, zuverlässigeren Alternativen deutlich gemacht. Ultraviolettes C-Licht (UV-C) bietet eine nicht-chemische, rückstandsfreie Methode zur Inaktivierung von Mikroorganismen, aber die Leistung handelsüblicher UV-C-Geräte variiert stark aufgrund von Unterschieden in Design, Leistung und Benutzerfreundlichkeit.

Im Review werden das derzeitige Spektrum an UV-C-Desinfektionstechnologien einschließlich Handgeräten, mobilen Robotern, statischen Gehäusen, in HVAC (Heizung, Belüftung und Klimatisierung) integrierte Systeme und an der Decke montierten Geräten bewertet und ihre Stärken, Grenzen und idealen Anwendungsfälle hervorgehoben. Unter diesen Geräten erweist sich das mobile UV-C-Desinfektionsgerät UVCeed als Lösung der nächsten Generation, die künstliche Intelligenz, verbesserte Realität und automatische Dosierung nutzt, um eine krankenhausgerechte Keimreduzierung mit unübertroffener Mobilität, Sicherheit und Benutzerfreundlichkeit bieten. UVCeed wurde unabhängig validiert, um bakterielle Krankheitserreger in weniger als einer Minute um >6 lg und virale Surrogate um >4 lg zu reduzieren und bietet damit eine skalierbare, kostengünstige und intelligente Alternative zu herkömmlichen Desinfektionsverfahren. Das Review positioniert UVCeed als Maßstab für eine moderne, zugängliche und effektive Oberflächendekontamination in einer zunehmend hygienebewussten Welt.

## Introduction

Ensuring clean and pathogen-free surfaces remains a cornerstone of infection control across clinical, commercial, and residential environments [1]. From hospital operating rooms to public transportation and household kitchens, contaminated surfaces are persistent vectors for microbial transmission, capable of harboring bacteria, fungi, and viruses for extended periods [2]. Traditional cleaning methods have relied heavily on chemical disinfectants, particularly quaternary ammonium compounds (QACs) and their non-QAC counterparts, such as alcohols, chlorine compounds, hydrogen peroxide, and phenolics [3], [4]. While chemically effective, these agents present major limitations in terms of safety, material compatibility, operational burden, and environmental sustainability [4], [5], [6].

The QACs, which account for more than a third of the United States (U.S.) disinfectant market, are widely recognized for their surface compatibility and residual antimicrobial activity [4]. However, increasing evidence links QACs to serious health risks, including occupational asthma, reproductive toxicity, and antimicrobial resistance [7], [8]. These concerns, coupled with their persistence in environmental systems, have prompted increased regulatory scrutiny and a gradual shift toward alternative chemical disinfectants. Yet, these alternatives – ranging from peracetic acid to hydrogen peroxide and alcohol-based products – are not without their own drawbacks [9], [10], [11]. Many are corrosive, flammable, or respiratory irritants, and nearly all require specific conditions such as surface wetness, adequate ventilation, and prolonged contact time to be effective [12], [13]. As a result, improper application remains a widespread issue, rendering even the most potent agents ineffective under real-world conditions.

Moreover, the ecological impact of these chemical disinfectants cannot be overlooked [14], [15], [16].Chlorine-based products can form toxic halogenated byproducts upon entering wastewater systems, while phenolic compounds persist in ecosystems, disrupting aquatic life and contributing to endocrine disruption [17], [18]. QAC [19] and even alcohols, often perceived as benign, contribute to indoor air pollution through volatile organic compound (VOC) emissions when used in poorly ventilated circumscribed areas such as incubators for premature neonates [20]. In parallel with these chemical concerns, there has been a growing recognition that a safer, faster, and more sustainable approach to disinfection is urgently needed – one that does not compromise efficacy for convenience or safety for speed.

In this context, ultraviolet-C (UV-C) disinfection has emerged as a powerful, non-chemical modality capable of addressing many of the limitations inherent to chemical-based solutions [21], [22]. Operating within the 200 to 280 nm range, UV-C light effectively inactivates pathogens by damaging nucleic acids and preventing replication [22]. Unlike chemical agents, UV-C leaves no residue, does not rely on consumables, and is less prone to user error when applied with the appropriate technology [2], [23]. Unlike chemical disinfectants, which pose risks of respiratory irritation and systemic absorption through inhalation, UV-C disinfection, when properly shielded, avoids these hazards entirely, making it particularly advantageous in sensitive environments such as operating rooms, pediatric care, and food preparation areas. Its efficacy has been well established across a spectrum of microorganisms, including enveloped viruses like SARS-CoV-2, antibiotic-resistant bacteria, and fungal spores [2].

The UV-C technology boasts a wide range of available products, spanning from low-cost consumer wands to complex hospital-grade robots and heating, ventilation, and air-conditioning (HVAC) UV-C units [23]. The performance of these devices varies dramatically based on factors like wavelength output, exposure time, surface distance, device geometry, and user technique [21], [24]. Many products lack any feedback or safety systems, leaving efficacy largely in the hands of the operator. While UV-C devices as a class offer tremendous potential, the absence of standardization or regulation has created a marketplace flooded with devices that differ significantly in their ability to deliver consistent and meaningful pathogen reduction [25].

Amid this variability, one device has emerged that effectively bridges the gap between clinical-grade performance and everyday usability: the UVCeed Mobile UVC Disinfection Device (UVCeed LLC, Effingham, Illinois) [23], [26]. Leveraging augmented reality (AR), artificial intelligence (AI), and intuitive user interfaces, UVCeed delivers a comprehensive, interactive, and verifiable disinfection process. It has been independently validated to achieve >6 lg reductions of bacterial pathogens such as *Staphylococcus (S.) aureus* and *Escherichia (E.) coli*, and >4 lg reductions for SARS-CoV-2 within 64 seconds, outperforming both traditional UV-C and chemical benchmarks [23], [25]. Unlike traditional UV-C wands, which rely on the user’s judgment to determine distance, angle, and dwell time, UVCeed integrates real-time AR overlays and AI algorithms that dynamically optimize exposure based on environmental conditions and device movement [27]. This ensures not only effective pathogen inactivation but also consistent and reproducible disinfection, irrespective of user experience. Furthermore, UVCeed incorporates critical safety features such as motion detection that automatically pauses UV-C output in the presence of nearby humans or pets [1]. Its compact, lightweight design and smartphone compatibility make it uniquely positioned for a broad range of use cases – from hospital operating rooms and outpatient clinics to schools, restaurants, and homes [23]. 

In an increasingly hygiene-conscious world, the demand for disinfection solutions that are safe, fast, effective, and environmentally sustainable has never been greater. The UVCeed answers this call not merely as another UV-C tool, but as a next-generation platform, combining the intelligence of modern computing with the power of germicidal light to redefine the standards of surface disinfection. The aim of this manuscript is to summarize and collate available UV-C technology, summarizing the different types (handheld UV-C wands, UV-C robots, static UV-C enclosures, and HVAC UV-C systems) available while comparing/contrasting their advantages and disadvantages.

## Handheld UV-C wands

Handheld UV-C devices are among the most accessible disinfection tools (Table 1 (Tab. 1)), widely marketed to both consumers and clinical users for their portability and affordability [28]. These devices, which resemble flashlights or wands, are typically used to disinfect high-touch surfaces, mobile electronics, and small workspaces [29]. Their appeal lies in their simplicity – users can wave the device over a surface to irradiate pathogens with germicidal UV-C light [30]. However, this simplicity can also be their greatest limitation. Most consumer-grade wands lack integrated dosage control, feedback mechanisms, or safety systems, placing the burden of efficacy entirely on the user. The result is often inconsistent exposure, missed areas, and suboptimal disinfection. Moreover, many devices fail to achieve significant microbial reductions under real-world use conditions [31]. While some may claim 99.9% efficacy, testing often reveals modest 2 to 3 lg reductions at best, with performance heavily dependent on proximity, angle, and dwell time. Some also pose safety risks due to unshielded UV-C emission, which can be harmful to skin and eyes if directed improperly [24], [32].

That said, a new generation of handheld UV-C wands – led by UVCeed – addresses these shortcomings through advanced automation and smart technology. The UVCeed incorporates AI-guided dosing, augmented reality (AR) visualization, and proximity-based control, ensuring optimal exposure with minimal effort or training [23]. In independent studies, UVCeed consistently achieved >6 lg reductions in bacterial load and >4 lg reductions of SARS-CoV-2 within one minute, while also pausing operation in the presence of people or pets [25]. This positions UVCeed not just as a handheld wand, but as a portable, intelligent disinfection platform, elevating handheld UV-C from an inconsistent option to a hospital-grade solution.

## UV-C technologies

Among the broad array of handheld UV-C disinfection technologies on the market, UVCeed has a host of unique advantages, including safety compliance, portability, smart features, and overall price, making it the most advanced and effective solution [23]. Unlike traditional wands that require manual dosing, fixed exposure times, and carry variable efficacy, UVCeed employs AI-driven smart dosing, surface recognition, and real-time safety protocols, ensuring optimal microbial reduction with minimal user intervention [33]. Clinically validated to achieve >6 lg reduction of pathogens such as methicillin-resistant Staphylococcus aureus (MRSA) and *E. coli* and a greater than 4 lg reduction of SARS-CoV-2 surrogates <1 minute, UVCeed offers hospital-grade disinfection in a compact, affordable, and portable format [25]. Its unmatched combination of efficacy, automation, and safety positions it not only as the superior choice in the handheld category, but also as a compelling alternative to bulkier, far more expensive UV-C systems. 

The UV-C disinfection robots and mobile towers represent the most powerful and autonomous class of UV-C technology [34]. These devices, typically costing tens to hundreds of thousands of dollars, are used primarily in hospitals, airports, and other institutional environments. Products like Xenex LightStrike (Xenex Disinfection Services, San Antonio, TX), Tru-D SmartUVC (PDI Healthcare, Woodcliff Lake, NJ), and R-Zero Arc (R-Zero Systems, Salt Lake City, UT) are designed for room-scale disinfection, emitting high-intensity UV-C light in 360° to sanitize walls, floors, and equipment [30]. The main strength of these systems lies in their broad-area coverage and automation. Many are equipped with sensors and mapping software that help them navigate spaces and calculate optimal exposure times. Clinical studies have shown that they can reduce bacterial loads by 4 to 6 lg [35].

However, these systems also face substantial logistical and financial barriers [36]. Due to their intensity, they must only be operated in unoccupied rooms, making their use episodic rather than continuous [37]. Their size and weight limit deployment to large, structured environments. Moreover, high upfront costs, maintenance needs, and training requirements make them inaccessible for smaller facilities, outpatient clinics, or home environments [38]. By contrast, UVCeed offers a portable alternative that rivals the efficacy of robotic towers, with validated performance in laboratory conditions and none of the operational constraints.

## Static UV-C enclosures (boxes and cabinets

The UV-C enclosures are static, enclosed devices designed to disinfect small items such as mobile phones, keys, ID badges, or stethoscopes. Popular examples include CleanSlate UV (CleanSlate UV, Toronto, Canada), PhoneSoap Pro (PhoneSoap LLC, Lehi, UT), and Coral UV (Coral UV, Markham, Canada) [39]. These units provide a controlled environment, allowing UV-C light to reflect and irradiate the contents from multiple angles without user exposure risk [40], [41]. The primary advantage of static UV-C boxes lies in their safety and reproducibility. Because the interior environment is controlled and isolated, the disinfection process is uniform and predictable [42]. This makes them useful in hospitals for disinfecting personal electronics or in food service for cleaning utensils and packaging.

However, their small internal capacity and inability to disinfect larger surfaces or environments limit their utility [42]. They cannot be used on complex or fixed surfaces, nor do they offer any flexibility in form factor. Additionally, they typically do not incorporate smart features like usage tracking or exposure validation, which limits their integration into broader infection control programs [41]. 

## UV-C disinfection for water bottles and liquids

The UV-C disinfection has become increasingly popular in personal hydration systems, particularly through self-cleaning water bottles equipped with integrated UV-C LEDs [43]. Products like the Larq Bottle by Brita (Taunusstein, Germany) are designed to inactivate bacteria, viruses, and biofilms in drinking water, providing users with a convenient, chemical-free method to ensure microbiological safety, especially in travel or uncertain water environments. These systems typically claim disinfection within 60 seconds [43]. However, quality and performance can vary significantly across brands. Many inexpensive or unregulated models lack verified dosing, shielding, or third-party efficacy data, raising concerns about user safety and reliability under real-world conditions [44]. 

In contrast, UV-C offers a non-chemical, residue-free, and consumable-safe alternative for disinfecting both fluids and surfaces. In addition to surfaces and devices, UV-C disinfection may hold promise for decontaminating reusable siphon pumps and other small-volume water transfer tools in hospital settings, especially when chemical exposure is undesirable or impractical. With appropriate surface access and dosage control, portable systems could in theory offer a non-chemical, residue-free solution to improve hygiene in fluid-handling processes. Notably, the UVCeed system includes a proprietary lid adapter that attaches to most standard reusable water bottles, enabling disinfection of both the internal contents and the external surfaces, specifically the mouthpiece and grip areas where lips and hands commonly transmit microbes. This dual-action capability enhances hygiene, particularly in healthcare, public, and travel settings where contamination risks are elevated.

## HVAC-integrated UV-C systems

The UV-C systems integrated into HVAC infrastructure serve a different purpose: airborne pathogen mitigation [45]. These units, such as those from Sanuvox (Sanuvox Technologies Inc., Montreal, Canada) or BioShield UV-C (BioShield Technologies Inc., Atlanta, GA), are placed inside air ducts or mounted near coils and vents to continuously inactivate pathogens in circulating air [45]. Their main role is in reducing airborne transmission, particularly in high-traffic or high-occupancy settings like hospitals, schools, and office buildings [46]. A strength of these systems is their continuous, passive function, operating without user input and covering large volumes of air. Properly maintained, they can decrease pathogen loads in HVAC systems and improve indoor air quality. However, they do not address surface contamination, and their efficacy can be difficult to verify without specialized testing [46]. Installation costs, airflow considerations, and maintenance requirements also limit their scalability in smaller or older buildings [47]. While HVAC UV-C complements other disinfection modalities, it cannot substitute for surface decontamination, especially in environments where fomite transmission plays a key role.

## Ceiling- or wall-mounted UV-C fixtures

Mounted UV-C systems are designed to deliver ambient disinfection in occupied spaces, often through indirect or shielded UV-C exposure [48]. Brands such as UV Angel (UV Partners Inc., Grand Haven, MI) and Violet Defense (Violet Defense Group, Orlando, Florida) are often found in lobbies, restrooms, or breakrooms, providing passive, continuous microbial suppression [49]. These systems work by irradiating air or surfaces over prolonged periods at safe intensity levels [50]. Their main advantage is occupancy-safe operation, enabling around-the-clock disinfection without disrupting workflow. However, their efficacy depends on prolonged exposure and optimal placement, making them less suited for rapid or deep surface decontamination [49]. Additionally, the fixed nature of these systems prevents flexible deployment and limits coverage to pre-installed zones. These systems are best used as adjunctive tools, maintaining baseline microbial control in spaces with moderate contamination risk [51]. For high-touch or rapidly contaminated areas, portable systems like UVCeed deliver faster and more complete disinfection, with the added benefits of real-time tracking, smart guidance, and surface-specific optimization (Table 2 (Tab. 2)).

## Discussion

The UV-C disinfection landscape encompasses a broad spectrum of technologies, each with distinct use cases, advantages, and limitations [52]. From autonomous UV-C robots designed for hospital-scale disinfection to compact static enclosures for sanitizing personal items, the diversity of available platforms underscores the versatility and complexity of UV-C as a disinfection modality [53]. However, this variability also makes direct comparisons challenging, particularly given the lack of uniform performance metrics and regulatory oversight.

### Technology and automation 

Most traditional UV-C systems rely on manual operation or static exposure times, which introduce variability in dosing and inconsistent coverage [39], [54]. Robotic systems like Xenex LightStrike and Tru-D SmartUVC incorporate automation and mapping software, but they are constrained by room size, cost, and operational inflexibility. In contrast, static enclosures such as CleanSlate UV and PhoneSoap offer reproducible exposure within a fixed chamber but are inherently limited to small, enclosed objects [45].

By comparison, UVCeed introduces a major technological leap in UV-C disinfection. Its AI-guided control, surface recognition, and real-time augmented reality (AR) interface ensure accurate dosing and thorough coverage across irregular, complex, or mobile surfaces [23]. Unlike other handheld wands [55], which leave efficacy largely to user judgment, UVCeed’s smart system automates the process, adapting UV-C intensity and exposure duration based on surface type, distance, and motion. Because UV-C requires direct line-of-sight to be effective, devices lacking real-time visual feedback risk incomplete disinfection [56]. The UVCeed’s integrated camera and AR interface are critical advantages, allowing users to visualize treated areas and ensure comprehensive, accurate coverage – something traditional UV-C devices cannot reliably achieve. Furthermore, when considering UV-C efficacy diminishes rapidly with distance, accurate dosing depends on precise calculation of light intensity, exposure time, and proximity [23]. Most UV-C devices rely on reflected light, which significantly reduces actual surface exposure and creates a false sense of disinfection, often compensated for by applying excessive, uncontrolled doses. UVCeed uniquely addresses this through real-time dose control – using visual and distance sensors, motion tracking, and onboard computation to precisely calculate and deliver the optimal UV-C energy, avoiding both underdosing and the risks of surface damage from overexposure. UVCeed’s integrated distance sensor and computational algorithms ensure exact dosing, while devices lacking these features rely on rough estimates, resulting in inconsistent and potentially ineffective disinfection. This combination of AI, AR, and gamified user feedback remains unparalleled in the UV-C market, making UVCeed the only system that merges flexibility with intelligent automation.

### UV-C dose control: The problem with distance and “dumb” devices 

The effectiveness of UV-C disinfection is highly dependent on dose, which is a function of light intensity, exposure time, and distance [23]. As distance increases, the time required to deliver an effective dose rises exponentially. For example, to achieve a commonly used target energy of 20 mJ/cm² for inactivating *E. coli*, a typical handheld UV-C wand would require [25]:

- 3.9 seconds at 2.5 inches (6.35 cm)
- 15.75 seconds at 5 inches (12.7 cm)
- 63 seconds at 10 inches (25.4 cm)

These calculations highlight a critical limitation of most handheld devices, which operate without visual sensors, distance measurement, or intelligent feedback, leading to inconsistent or incomplete disinfection. By contrast, UVCeed continuously monitors motion and distance using its integrated camera and sensors, adjusting exposure in real time to ensure consistent and effective UV-C dosing [23]. 

### Efficacy and lg reduction 

When evaluating pathogen inactivation, lg reduction values are critical. The UV-C robots, such as R-Zero Arc and Xenex LightStrike, have demonstrated 4 to 6 lg reductions in controlled settings, primarily under ideal, unoccupied room conditions [31]. Mounted and HVAC-integrated systems offer continuous background disinfection, but their log reduction data is often limited, variable, or based on surrogate pathogens under prolonged exposure.

The UVCeed, however, delivers >6 lg reduction of MRSA and *E. coli* and more than 4 lg reduction of SARS-CoV-2 surrogates within 64 seconds at clinically relevant distances [25]. These outcomes are validated under real-use conditions, making UVCeed’s efficacy not just theoretical but practically reliable across a wide range of environments – from operating rooms to outpatient clinics, classrooms, and households. Of note, demonstrated efficacy against non-enveloped viruses has not yet been established.

### Cost and accessibility 

One of the most striking differences between UV-C technologies lies in cost-effectiveness. Full-scale disinfection towers and robots typically range from $30,000 to over $125,000, requiring dedicated staff, room downtime, and integration with building systems [45]. Static boxes and mounted units, while more affordable, are highly task-specific and lack broader utility. By contrast, UVCeed retails at just $129.95 yet delivers performance comparable to, or exceeding, that of hospital-grade systems. Its low price point, combined with portability and smart technology, enables widespread adoption in both professional and personal settings. 

### Safety and compliance 

Chemical disinfectants like QACs and alcohols are limited to hard, nonporous surfaces and are unsafe for use around food or liquids, often requiring four or more minutes of continuous wet contact to be effective [4]. QACs leave behind toxic residues, can cross the blood-brain barrier, causing CNS damage (notably to oligodendrocytes, as shown in recent studies), and offer no visibility into whether the correct dose was applied or if surfaces were fully treated [5]. 

Many UV-C products, especially lower-cost wands, pose safety risks due to direct exposure to UV-C radiation, lack of shielding, and absence of proximity sensors. The HVAC and mounted systems, while safer, offer little transparency regarding their effectiveness or coverage [45]. The UVCeed incorporates machine vision and safety interrupts that pause operation when human or pet presence is detected. This is coupled with no ozone production, real-time AR safety cues, and dosage optimization that avoids surface degradation [23]. These features establish a new safety benchmark for UV-C use in both clinical and public environments.

The majority of UV-C devices are “dumb” – unable to see what has or hasn’t been disinfected, nor can they calculate the actual dose applied, making efficacy a guess. The UVCeed is the only UV-C system combining a camera, sensors, and AI algorithms to actively prevent UV-C exposure to humans, pets, or unintended objects, setting a new standard for safety [23]. Beyond bacteria and viruses, UV-C effectively inactivates mold and fungi, which are major contributors to respiratory illness, allergic reactions, odors, and food spoilage [25]. The UV-C technology is already widely used in food prep, packaging, water treatment, laboratories, biosafety cabinets, and hospital environments, demonstrating its broad utility, provided that safety, efficacy, and precise dose control are maintained, as exemplified by UVCeed.

### Limitations 

Despite the advantages outlined in this review, several limitations warrant consideration. Currently, there is no available efficacy data for UVCeed or similar UV-C systems against non-enveloped viruses, which are known to be more resistant to environmental interventions than their enveloped counterparts. Additionally, while preliminary observations suggest that UVCeed can deliver surface disinfection more rapidly than conventional wipe-based methods – particularly by eliminating the need for wet contact time and post-application drying – a direct, time-matched comparison has not yet been formally conducted. A dedicated study is underway to evaluate these performance differences in real-world clinical settings. Finally, as with all UV-C devices, effective disinfection depends on proper exposure, including adequate proximity, dwell time, and unobstructed access to target surfaces. Surfaces obscured by geometry, residue, or user error may receive subtherapeutic dosing unless compensated for by advanced guidance technologies like those integrated into UVCeed.

## Conclusions

The landscape of surface disinfection has long been dominated by chemical agents and bulky institutional UV-C systems, each with inherent limitations in efficacy, usability, cost, or safety. In contrast, UVCeed represents a paradigm shift, offering a smart, portable, and clinically validated solution that delivers hospital-grade disinfection with unmatched user accessibility. Through the integration of augmented reality, artificial intelligence, and real-time safety monitoring, UVCeed transforms UV-C light from a static technology into a dynamic platform capable of adapting to diverse environments and user needs. Its superior log reduction performance, intuitive design, and affordability make it not just a superior handheld device, but a compelling alternative to traditional UV-C and chemical disinfection across clinical, commercial, and personal settings. As hygiene expectations rise and disinfection practices evolve, UVCeed sets a new benchmark, redefining what is possible in rapid, reliable, and responsible pathogen control.

## Notes

### Competing interests

Ng MK has received consulting fees from Johnson & Johnson Ethicon, Pacira BioSciences, Inc., Sage Products, Inc., Bonutti Technologies Inc., Hippocrates Opportunities Fund LLC, and Ferghana Partners, Inc. 

Mont MA has received consulting fees from 3M, Johnson & Johnson, Smith & Nephew, Pacira BioSciences, Inc., and Stryker; research funding from the National Institutes of Health and Stryker; is a shareholder for CERAS Health, Peerwell, and MirrorAR; serves as a board member for the Hip Society and the Knee Society; and is the Editor-in-Chief for The Journal of Arthroplasty, and an editor for the Journal of Knee Surgery, Surgical Technology International, and Orthopaedics. 

### Authors’ ORCIDs

- Ng MK: https://orcid.org/0000-0002-5831-055X
- Mont MA: https://orcid.org/0000-0003-4303-5556

### Funding

No funding received for this project

## Figures and Tables

**Table 1:**
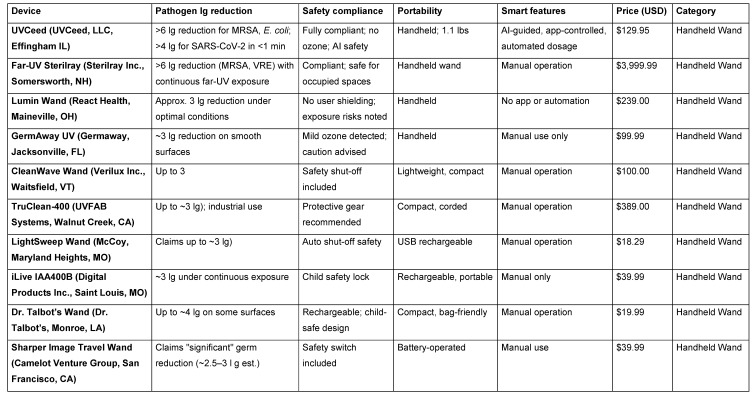
Summary of available UV-C handheld wand technology

**Table 2:**
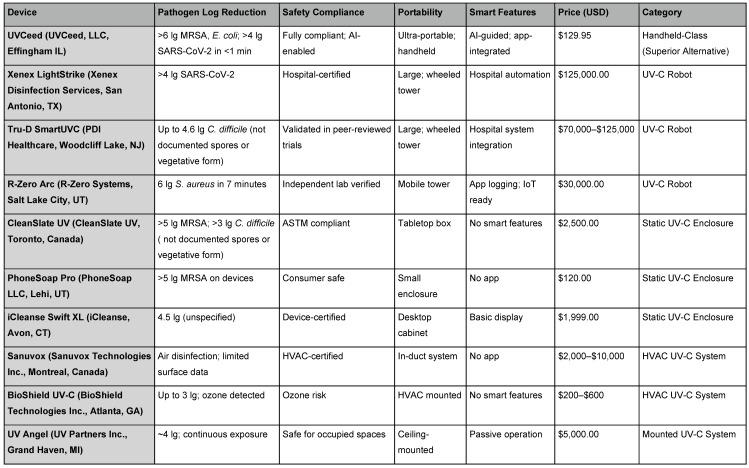
Summary of available non-handheld UV-C technologies
